# Equity in development and access to health services in the Wild Coast of South Africa: the community view through four linked cross-sectional studies between 1997 and 2007

**DOI:** 10.1186/1472-6963-11-S2-S5

**Published:** 2011-12-21

**Authors:** Steven Mitchell, Neil Andersson

**Affiliations:** 1 CIETcanada, 1 Stewart Street, Ottawa, Ontario, Canada; 2 Centro de Investigación de Enfermedades Tropicales, Universidad Autónoma de Guerrero, Acapulco, Mexico

## Abstract

**Background:**

After election in 1994, the South African government implemented national and regional programmes, such as the Wild Coast Spatial Development Initiative (SDI), to provoke economic growth and to decrease inequities. CIET measured development in the Wild Coast region across four linked cross-sectional surveys (1997-2007). The 2007 survey was an opportunity to look at inequities since the original 1997 baseline, and how such inequities affect access to health care.

**Methods:**

The 2000, 2004 and 2007 follow-up surveys revisited the communities of the 1997 baseline. Household-level multivariate analysis looked at development indicators and access to health in the context of inequities such as household crowding, access to protected sources of water, house roof construction, main food item purchased, and perception of community empowerment. Individual multivariate models accounted for age, sex, education and income earning opportunities.

**Results:**

Overall access to protected sources of water increased since the baseline (from 20% in 1997 to 50% in 2007), yet households made of mud and grass, and households who bought basics as their main food item were still less likely to have protected sources of water. The most vulnerable, such as those with less education and less water and food security, were also less likely to have worked for wages leaving them with little chance of improving their standard of living (less education OR 0.59, 95%CI 0.37-0.94; less water security OR 0.67, 95%CI 0.48-0.93; less food security OR 0.43, 95%CI 0.29-0.64). People with less income were more likely to visit government services (among men OR 0.28, 95%CI 0.13-0.59; among women OR 0.33, 95%CI 0.20-0.54), reporting decision factors of cost and distance; users of private clinics sought out better service and medication. Lower food security and poorer house construction was also associated with women visiting government rather than private health services. Women with some formal education were nearly eight times more likely than women with no education to access health services for prevention rather than curative reasons (OR 7.65, 95%CI 4.10-14.25).

**Conclusion:**

While there have been some improvements, the Wild Coast region still falls well below provincial and national standards in key areas such as access to clean water and employment despite years of government-led investment. Inequities remain prominent, particularly around access to health services.

## Background

In 1994 the new South African government declared the overall priority of eradicating poverty and removing *inequities* - socioeconomic inequalities and differential access to services that are unfair or unjust [1]. As a result, the government created the Reconstruction and Development Programme (RDP) to reduce poverty and distribute income more evenly. Less spending was to go to the military, and more was to be distributed on education, housing, and health, including the building and upgrading of clinics and promises of free health care to children under six and pregnant mothers [2]. The RDP however, fell out of public view within two years, and the ministry overseeing it was abolished [3]. It was criticized by some as a short-sighted programme of basic needs fulfilment [4].

In 1996, to help meet the goals of the RDP and respond to neoliberal influences, the government adopted the Growth, Employment & Redistribution (GEAR) macroeconomic policy. GEAR intended to reduce the role of the state and increase corporate and private investment [5,6]. GEAR was publicly proposed as a way to provide a fast growing economy, create jobs, redistribute income, and hasten universal access to basic needs [7]. Consistent with a focus on decentralization, the national, provincial, and local governments adopted local economic development (LED) strategies which aimed to reduce poverty and increase employment through local initiatives and solutions [8]. LED encourages communities to take control to stimulate economic growth through community-based initiatives and local skills, resulting in increased opportunities, community empowerment and self reliance [9].

One of the policies adopted along with LED was the creation of Spatial Development Initiatives (SDIs) in 1997 by the South African Department of Trade and Industry (DTI), intended to promote and encourage private investment and development in areas that were considered to have the greatest potential for growth. The SDIs focussed on short term interventions designed to attract private sector investment, to stimulate growth of locally owned small, medium and micro-enterprises (SMMEs), and empower local communities [10]. They identified and sought to address bottlenecks to investment, such as inadequate infrastructure (water, roads, electricity, and communications) [11]. Development was concentrated in relatively small areas rather than thinly spread across larger regions or provinces [5]. The SDIs expected to benefit rural communities through increased employment, improvement of local infrastructure, and income from leasing out lands [12].

Since 1994, there have been some successes nationally as a result of the national and local development strategies. These include new health clinics, schools, housing, and improved water facilities [2]. Yet there are also reports that many South Africans have become disillusioned at the lack of progress, particularly with regards to standard of living and employment. For example, unemployment rates rose from 19% in 1996 to 29% in 2001 [13]. Economic growth rates have been modest at best and South Africa is often seen as one of the most unequal societies with regards to distribution of income [14]. Some authors have described a dual economy, the “first economy” containing the industrial, mining and agricultural sectors that produce wealth, while the “second economy” is characterised by poverty and underdevelopment [15-17].

Concerns exist around health care as well. Many South Africans criticize the government’s handling of the HIV/AIDS epidemic [18], one of the leading causes of life expectancy estimates declining by ten years between 1996 and 2002 [19]. Furthermore, public spending is rarely optimised towards the poor [20,21]. The wealthiest provinces receive most health care expenditure, and since 1999 there has been increasing emphasis on privatized health care which the poorer regions cannot afford [22]. This leaves the most vulnerable less likely or unable to access health services when they need them, leading to higher risk of poor health, increasing the burden of future health costs, and reducing their ability to seek employment or farm their own lands [23,24].

**Figure 1:**
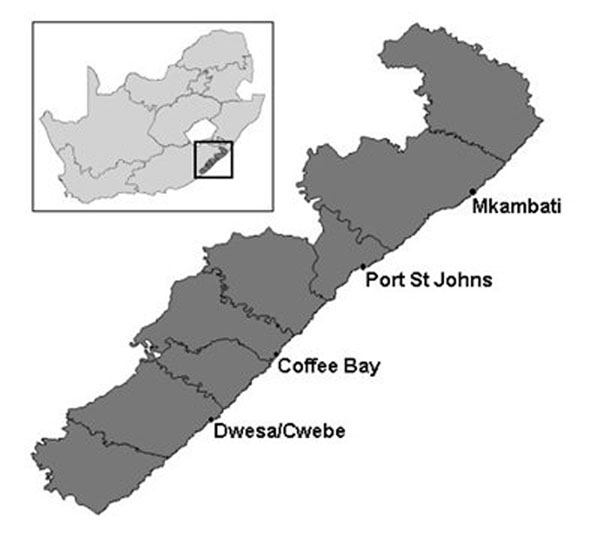
The Wild Coast region of the Eastern Cape Province, showing anchor locations

The Eastern Cape is one of nine provinces in South Africa and is located along its south eastern shore. It was formed in 1994 with the new government, encompassing the previously Transkei and Ciskei Xhosa homelands. It is one of the poorest provinces in the country and by 2001 only one-fifth of the population was employed [25]. It has two large cities - Port Elizabeth and East London - but much of the region is rural and relies on subsistence farming. The province is home to the Wild Coast region, located along its north eastern coast (Figure 1). After years of labour migration under the apartheid system, by 1994 the Wild Coast population was predominantly female and unemployed. At that time the Wild Coast had little access to clean water or public service infrastructure. Unemployment was higher than the national average and much like the province as a whole; nearly three-quarters of the Wild Coast population lived in poverty [4]. The region also faces many health threats including HIV/AIDS and tuberculosis [26].

The Wild Coast SDI started in 1997 with a particular focus on tourism and SMME development. Agriculture and forestry were other sectors that were identified to stimulate growth, with private companies partnering with communities [12]. Such initiatives expected to create economic opportunities for local populations, particularly women. The SDI identified four coastal high potential “anchor” areas as the focus for public and private investment: Mkambati, Port St Johns, Coffee Bay and Dwesa/Cwebe. SDI planners felt that intensive investment in these four areas would spill out and spur economic development in the rest of the Wild Coast region.

In partnership with the Eastern Cape Socio-economic Consultative Council (Ecsecc), CIET assessed the Wild Coast SDI over several years. While coverage of basic needs (such as water and health) was not an explicit goal of the SDI, early feedback from community-based evaluation of the SDI showed that unless these were met the initiative had little chance of success. A 1997 baseline study showed that people in the region were unaware of what they could do to improve their socio-economic conditions. There were high levels of unemployment and lack of food security, a low proportion of households obtained their water from protected sources such as taps, there was a substantial degree of corruption in the public services (including health), and little knowledge of the SDI project itself [27]. Follow-up surveys in 2000 and 2004 showed little evidence of increased economic opportunities [28,29].

The Wild Coast SDI was terminated since the 2004 evaluation. Responsibility for development of the region moved from the DTI to the Department of Environmental Affairs and Tourism (DEAT). Newer initiatives in the area have included the EU community-based tourism initiative, the controversial N2 toll road, the establishment of the Pondoland National Park and a new Wild Coast Development Project. It remains unclear how these new initiatives intend to decrease poverty and improve health in the region, as they seem poised to repeat the shortcomings of the SDI. An additional 2007 follow-up survey of the same communities provided an opportunity to examine the inequities detected in the original 1997 baseline (such as access to clean water, food security, household construction, education and employment), and how such inequities affect access to health care.

## Methods

The methods relied on standard CIET social audit protocols [30,31]. We stratified the last stage random sample of twenty communities by anchor/non-anchor status, geographic location (such coastal/non-coastal), proximity to infrastructure, and road accessibility. The 2000, 2004 and 2007 follow-up surveys returned to the sites of the 1997 baseline. Data collection instruments across the different cycles included household questionnaires and community profiles. We additionally shared and discussed preliminary findings with the participating communities through gender stratified focus groups. We translated all instruments into isiXhosa and then non-members of the research team translated them back into English to ensure questions remained true to their intended meaning. We piloted the instruments extensively before implementing them in the field to refine the instruments, test for clarity and ensure proper translation.

The CIETinternational ethical review board conducted and granted ethical clearance. Fieldworkers recorded and stored household data without any identifying fields, ensuring confidentiality of the respondents. We maintained confidentiality of the sample community identities as much as possible, especially with regard to the non-anchor areas. The exact sample sites were not included in any reporting.

Data entry and analysis relied on public domain software EpiInfo [32], and open source analysis and geomatics software CIETmap [33]. We adjusted indicators to account for the effect of uneven sampling, and report weighted results. We examined associations between factors in bivariate and then multivariate analysis using the Mantel Haenszel procedure [34]. Multivariate models took into account potential household inequities such as non-anchor status, household crowding, access to protected sources of water, roof construction, main food item purchased, and perception of community empowerment. Individual level models additionally accounted for age, sex, education and income earning opportunities. For access to health services, we made separate models for men and women; and limited these to those aged 18-65 in order to account for income earning opportunities.

We adjusted for clustering using a method produced by Gilles Lamothe based on a variance estimator to weight the Mantel Haenszel odds ratio for cluster-correlated data, described elsewhere [35]. We describe associations using the Odds Ratio (OR), indicating where this is adjusted by stratification (ORa), accompanied by the cluster adjusted 95% confidence interval (CIca). Averages are accompanied by a measurement of the standard error (se) and the total number (n). We derived measurements of trend using the Mantel-Haenszel extension [36]. Some indicators were not comparable or collected in 1997, and for these trends compare 2000 to 2007.

We imputed ten additional datasets using the Amelia II program for missing data [37] to test how missing data would affect the final models. These tests showed little effect on the final models, so we report the original results.

## Results

### Socio-economic indicators

#### Household characteristics

In 2007, we collected data from 2401 households. Respondents provided information about 8496 individuals. Among these, 57% (4830/8478) were female, a nearly identical proportion to previous years. Average household size in 2007 was 3.7 people (SD 2.2, n2378), the same as in 2004 but lower than in 1997. One-third (777/2322) of households were made of mud with grass thatch roofs, a significant reduction from previous years (Table 1).

**Table 1:** Household characteristics – weighted % (fraction)

	1997	2000	2004	2007	χ^2^ trend (p)
households	2457	2363	2383	2401	-
non-anchor areas	71 (1598/2457)	72 (1552/2363)	71 (1560/2383)	72 (1579/2401)	0.204 (0.65170)
female respondents	71 (1736/2457)	78 (1817/2363)	75 (1781/2378)	73 (1726/2398)	0.303 (0.58174)
mud and grass thatch roof house construction	50 (1269/2455)	56 (1285/2342)	47 (1088/2374)	34 (777/2322)	190.250 (0.00000)
main food item purchased is basics	95 (2255/2399)	96 (2191/2297)	91 (2081/2279)	85 (1959/2317)	153.814 (0.00000)
average household size	5.8 (SD 3.0, n2457)	4.7 (SD 2.7, n2363)	3.7 (SD 2.3, n2384)	3.7 (SD 2.2, n2378)	-

#### Community empowerment - hearing about and having a say in development

When asked in 2007 what development projects respondents had heard about in their area, only one-quarter of household respondents could name something (500/2026). Only one respondent mentioned the SDI by name when asked about development projects in 2007. Among those who had heard of any development projects, only one half (246) felt they had a say in it.

#### Sources of water

**Figure 2:**
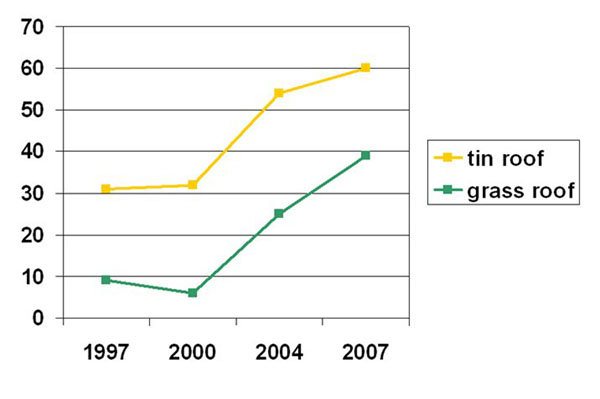
% of households with protected sources of water, by type of house construction

Just over half (1284/2359) of households in 2007 got their water from a relatively protected source, such as a tank or tap. Households made of mud and grass, and households who bought basics as their main food item were less likely to have protected sources of water (Table 2). There has been a significant and steady increase in households having access to protected sources of water since the baseline, from 20% (550/2455) in 1997 to 52% (1284/2359) in 2007 (χ^2^ trend 756.4, p=0.00000). The increase is consistent across different household types, for example, both among those with tin roofs and among those with grass roofs, yet inequities remain between the two (Figure 2).

**Table 2:** Final multivariate models of variables associated with development outcomes 2007

	ORa	95%CIca for adjusted OR
**Households with access to protected water**		
Mud and grass thatch roof house construction	0.38	0.21 – 0.71
Main food item purchased is basics	0.66	0.46 – 0.94
Worked for wages in the previous month*		
Female	0.63	0.52 – 0.75
No education	0.59	0.37 – 0.94
Main household food item purchased is basics	0.43	0.29 – 0.64
Mud and grass thatch roof house construction	0.7	0.55 – 0.89
Household with no protected water source	0.67	0.48 – 0.93
Owned their own business*		
Men	0.59	0.43 – 0.80
Men accessing health services in the last year*		
From a household with four or fewer people	1.39	1.02 – 1.89
From a household without loans	0.63	0.50 – 0.78
Women accessing health services in the last year*		
From a household with four or fewer people	1.36	1.07 – 1.72
From a household without loans	0.65	0.46 – 0.92
Men accessing a government or traditional health service*		
Income earning opportunity	0.28	0.13 – 0.59
From a household with a tin roof	0.61	0.38 – 0.98
Women accessing a government or traditional health service*		
Income earning opportunity	0.33	0.20 – 0.54
From a household with a tin roof	0.52	0.37 – 0.75
Main food item purchased is non-basics	0.48	0.37 – 0.75
Lives in anchor area	2.28	1.42 – 3.66
Men’s choice of facility based on better service, medication or referrals (not proximity, cost or no choice)*		
Has an income earning opportunity	2.37	1.43 – 3.96
No income from migrant workers	0.55	0.33 – 0.91
**Women attended health facility for preventative reasons (as opposed to curative reasons)**		
Had some formal education	7.65	4.10 – 14.25
No income from migrant workers	0.7	0.50 – 0.97
Women waiting less than one hour at govt services*		
Has an Income earning opportunity	0.68	0.48 – 0.97
From a household with four or fewer people	0.73	0.56 – 0.95
Main food item purchases is non-basics	0.53	0.32 – 0.89
From a household without loans	1.52	1.20 – 1.92

*among those aged 18-65

#### Food

In 2007, 85% (1959/2317) of households purchased basics such as maize as their main food item. The proportion purchasing basics was lower than in previous years (Table 1).

#### Employment and small business ownership

Some 21% (835/3737) of those aged 18-65 in 2007 worked for wages in the month prior to the survey. Women, those with no schooling, those who purchased basics as their main food item, those from households made of mud and grass thatch roofs, and those from households with unprotected sources of water were less likely to have worked for wages in the previous month (Table 2). There has been a gradual increase of those reporting having worked for wages over the years that we collected comparable data on this (2000: 15% 781/4852, 2004: 18% 821/4361; 2007: 21% 835/3737; χ^2^ trend 53.641, p=0.00000). Nearly 40% (717/1911) of households in 2007 received income from migrant workers, a slight increase from previous years (2000: 33% 724/2341; 2004: 34% 744/2211; χ^2^ trend 20.2561, p=0.00001).

Despite the objective of the SDI to generate small and medium economic activity, only 7% (282/4160) owned a business in 2007. Those from larger households, and men were less likely to own their own business in 2007 (Table 2). The proportion who owned a business was nearly identical to the proportions from previous years (Table 3).

**Table 3:** Small business ownership (among those 18-65)

	Weighted % (fraction) of respondents			χ^2^ trend (p)
	2000	2004	2007	
Ever considered starting their own business	31 (1491/4852)	29 (1278/4364)	32 (1257/4052)	0.041 (0.84039)
Currently owns	6 (319/4855)	8 (351/4398)	7 (282/4160)	0.257 (0.61240)

#### Household loans and credit

In 2007, some 16% (377/2230) of households had loans. This is the same proportion as in 1997 (417/2471) and 2004 (391/2256) but much lower than in 2000 (41%, 951/2302; χ^2^ trend 30.407, p=0.00000). There has been a significant increase of households reporting emergencies as the purpose of their loans, from 4% (13/408) in 1997, less than 1% in 2000 (2/917) and 2004 (1/388), to 13% (42/368) in 2007 (χ^2^ trend 49.425, p=0.00000). When asked about the source of their loan, 56% (204/364) claimed they got their loan from a loan shark, a source which has seen a dramatic and consistent increase since the baseline (1997: 2% 6/415, 2000: 2% 21/871, 2004: 35% 138/386; χ^2^ trend 570.469, p=0.00000).

### Access to health services

**Figure 3:**
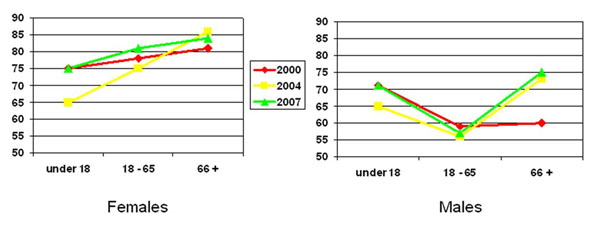
% who accessed health services within the last year (by age group and sex)

Accessed in the last year: access to health services by female residents increased each year among increasing age groups (under 18, 18-65, 66+). A lower proportion of male residents of working age (18-65 years) accessed health services than in the two other age groups in each year. Additionally, a lower proportion of those 18-65 accessed health care in 2007 than in 2000. Among those aged 66+, higher proportions of men accessed health services since 2000 (Figure 3). For both male and female residents aged 18-65, those from less crowded households (4 or fewer people) were more likely to have accessed health services in the last year; those from households without loans were less likely to have accessed health services in the last year (Table 2).

**Figure 4:**
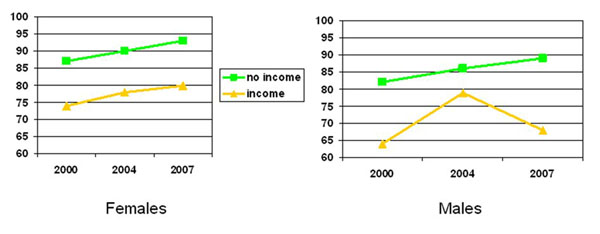
% who accessed government or traditional health services (as compared to private services) by income status (among those aged 18-65)

Type of health service used: There was an increase in the use of government services and a corresponding decrease in use of private services and hospitals, particularly among women (Table 4). Those with an income from wages or a business (Figure 4) and those from houses with tin roofs were less likely to have visited a government or a traditional health services (Table 2). Among female residents aged 18-65, those with an income earning opportunity, those from houses with tin roofs, and those who purchased non-basics as their main food item were less likely to have visited a government or traditional health service; and women from anchor areas were more likely to have visited a government or a traditional health service than those from non-anchor areas (Table 2).

**Table 4:** Type of health service visited in the last year (among those aged 18-65)

	Weighted % (fraction) of respondents		
Type of institution	2000	2004	2007
*men:*			

Government	60 (586/960)	68 (570/826)	67 (446/661)
Private	17 (150/960)	13 (106/826)	13 (93/661)
Traditional	1 (12/960)	1 (8/826)	2 (12/661)
Hospital	22 (212/960)	19 (142/826)	18 (110/661)

*women:*			

Government	69 (1556/2245)	72 (1490/2010)	76 (1350/1754)
Private	13 (284/2245)	11 (221/2010)	9 (169/1754)
Traditional	1 (5/2245)	1 (8/2010)	1 (12/1754)
Hospital	18 (400/2245)	16 (291/2010)	13 (223/1754)

#### Choice of health service

The most common reasons cited for choosing government health clinics were proximity, cost, and feeling there was no other choice. The most common reasons for choosing private clinics were good service, good medication, feeling there is no other choice, and referrals. Reasons for choosing government and private health services were nearly identical for male and female residents (Table 5). Among male residents aged 18-65 years, those with an income earning opportunity were more than twice as likely to choose the health institution on their last visit due to better service, better medication, or referrals (as compared to reasons such as proximity, cost, or lack of choice) than those without an income earning opportunity (Table 2). We found the same for female residents aged 18-65, but only among those who lived in houses that *did not* receive any income from migrant workers (ORa 2.47, CIca 1.55-3.95).

**Table 5:** Reasons for choosing type of health service in the last year, 2007 (among those aged 18-65)

	Weighted % (fraction) of respondents	
Reason	men	women
*Among users of government clinics:*		

Nearer	64 (272/421)	55 (732/1283)
Inexpensive/free	21 (86/421)	26 (298/1283)
No choice	8 (33/421)	12 (146/1283)
Good service	4 (15/421)	5 (63/1283)
Referral	2 (9/421)	2 (31/1283)
Good medication	2 (6/421)	1 (13/1283)

*Among users of private clinics:*		

Nearer	0 (0/74)	1 (2/139)
Inexpensive/free	1 (1/74)	1 (2/139)
No choice	20 (13/74)	17 (25/139)
Good service	54 (41/74)	45 (59/139)
Referral	10 (9/74)	12 (17/139)
Good medication	14 (10/74)	24 (34/139)

#### Attention needed

**Figure 5:**
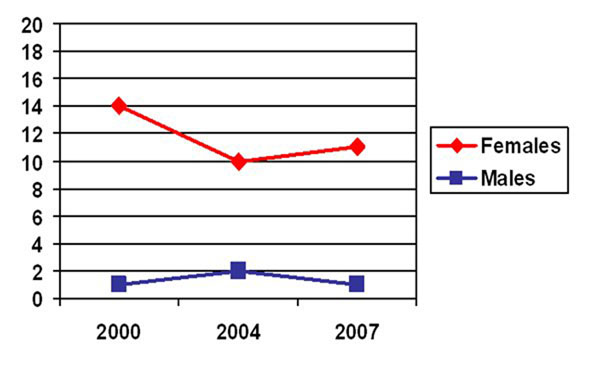
% who attended health service for prevention reasons (among those aged 18-65)

Some 8% (153/1781) of users of government clinics attended for prevention reasons like immunisation, while 2% (2/252) of users of private clinics attended for prevention reasons. A much lower proportion of male than female residents attended a health institution for prevention reasons – and only 5/642 men in 2007 attended for prevention reasons (Figure 5). Among female residents aged 18-65, those with some formal education were nearly eight times more likely to have accessed a government health service for prevention reasons than those with no formal education; and those who lived in households that received income from migrant workers were less likely to have accessed government health service for prevention reasons than those who lived in households that had not received income from migrant workers (Table 2).

#### Waiting times

Users of government facilities reported longer waiting times than users of private clinics; and female users overall reported longer waiting times than did male users in government facilities. However in 2007, female users of private clinics reported lower average waiting times than men (Table 6). Among male users (aged 18-65) of government clinics, those from households without loans were twice as likely to report waiting less than one hour for service than those from households with loans (ORa 2.06, 95% CIca 1.19-3.59). This was similar for female users as well (ORa 1.52, 95%CIca1.20-1.92). However, additionally among female users of government clinics, those with an income earning opportunity, those from less crowded households, and those whose main food item was not basics were *less* likely to have waited less than an hour for service (Table 2).

**Table 6:** Average waiting time (minutes) among those aged 18-65, by type of health service used

Year	Government		Private	
	men	women	men	women

2000	58 (se 2.8, n=493)	71 (se 2.3, n=1396)	36 (se 3.8, n=127)	59 (se 8.4, n=259)
2004	77 (se 5.6, n=484)	73 (se 2.1, n=1313)	50 (se 5.0, n=93)	52 (se 5.8, n=215)
2007	65 (se 3.4, n=414)	71 (se 2.5, n=1273)	49 (se 10.0, n=88)	34 (se 4.5, n=154)

#### Payments at government clinics

Payments at government clinics have decreased significantly overall since 2000 for both male and female health service users: only 2% (57/3432) of users of government clinics in 2007 claimed they paid something for their service on their last visit, fewer than in 2000 (26% 1159/5571) and 2004 (7% 201/4313) (χ^2^ trend 928.49, p=0.00000). We found no evidence of a difference by sex, age group or other socio-economic characteristics in the rare report of having made a payment in 2007.

## Discussion

The Wild Coast has seen development improvements since 1997, including increased access to protected sources of water and a marginal increase in employment. Pronounced inequities – such as differential access to health care based on education and income - were still evident in 2007.

### Water

Since the baseline in 1997, water supply from protected sources increased from 20% to 50% in 2007. Yet the proportion with access to protected sources in the Wild Coast region is lower than the provincial average (70%) and lower still than the national average (88%) [38]. This still leaves half of the population of the region without a protected source, making them susceptible to water-related illnesses such as diarrhoea and cholera. Reported improvements in access to water supplies in the Eastern Cape overall are offset by reports of poor water quality, particularly in rural areas [39,40], and it is possible that those with “protected” sources are not much better off than those without. As access to clean and safe water directly impacts on health and income potential, community and district capacities for ongoing and consistent monitoring and testing must be implemented alongside improved water infrastructure developments. Priority must also be given to ensuring water provision and quality in the Wild Coast increases to meet provincial and national standards.

### Income and employment

The SDI aimed to increase employment and to promote entrepreneurship. There has been no increase in the number of respondents considering owning their own business, or of those who actually do own a business. Employment levels among adults increased gradually from 15% in 2000 to 20% in 2007, but this still leaves a majority without work. As with water, employment rates within the region are still below the provincial and national rates [41] leaving the Wild Coast region largely in the same economic shape as before the initiative. Importantly, the most vulnerable (such as those with less education, and less water and food security) are less likely to have worked for wages, leaving them with little chance of improving their standard of living. Loan sharks have prospered as the main source of household loans. Increasing numbers of loans are to respond to household emergencies; few are for starting businesses or creating income opportunities.

### Access to health services

Fewer male than female residents accessed health services and, among those who did, very few did so for preventive reasons. Lower rates of men’s access may be explained by women's increased interaction with the health service through antenatal care. Yet other studies have found that men also tend to wait until they see signs of illness before seeking help or attention (such as testing for HIV) [35,42].

Striking differences in health care access exist between the most and least vulnerable within the region. Women with some formal education were nearly eight times more likely to access health services for prevention reasons, in comparison with those with no formal education. For both male and female residents, income was strongly related to type of health clinic visited, and the reason for doing so, consistent with results found in KwaZulu-Natal [43]. Those with less income were more likely to visit government services, reporting determinants of cost and distance; users of private clinics sought out better service and medication. Lower food security and poorer house construction was also associated with women visiting government, not private, health services.

Each of the male and female focus groups discussed a lack of satisfaction with government clinics, stressing poor service, and a lack of privacy as key concerns. Additionally, medication was reportedly either missing or expired, and several focus groups stated that patients were given “panado” regardless of their ailment. Average waiting times were also consistently lower for users of private clinics than for users of government clinics. Despite this, the proportion using government clinics increased. Payments at government health clinics for free services were nearly non-existent by 2007, an indication that corruption in the form of unofficial payments is no longer an issue. This is promising, as it frees up household resources for other needs. Focus groups still complain about favouritism among the nurses and doctors at the clinics, and removing user fees for service does not help those who need medicine that is unavailable.

Although unique as a detailed follow-up of health care and development in the Wild Coast, there are some limitations to this study. The cross sectional design only allows us to report associations and limits what we can conclude about causality. For example, when we state that those from households with unprotected sources of water were less likely to have worked for wages in the previous month, we cannot attribute causality in one direction or another.

Secondly, we can report with some confidence on trends over time, but we are unable to provide individual linkages through the years as one might through a longitudinal study that follows up with the same individuals in each year.

## Conclusion

The government’s economic and development initiatives since 1994 have failed in their short-term goals in the Wild Coast region, particularly with regards to employment and health. Policies such as the RDP and GEAR set out to improve quality of life, redistribute wealth in a more equitable manner, and increase economic activity in the most vulnerable areas. Yet much of the Wild Coast region was still without clean water in 2007 and the majority were unemployed. Much of the economic growth in the country as a whole since democracy has taken place in the larger urban centres, with smaller towns and rural areas falling further behind [44]. LED strategies aimed to stimulate growth locally and empower communities but there is little evidence of this happening in the Wild Coast, consistent with evidence nationally that suggests LED successes have been modest at best, and primarily located in larger well resourced cities [8]. The Wild Coast SDI sought to increase economic activity and foster the growth of SMMEs, yet there is no evidence of an increase in locally owned businesses or even the consideration of ownership. Furthermore, development initiatives seem to have failed in increasing access and improving health services, even though these were identified early on in the process as crucial for their success. By 2007, residents still complained of poor service and a lack of medications in government health clinics and there are still socio-economic inequities in terms of access, particularly for preventative reasons.

One might argue that development takes time and that the full effects of the initiatives have not yet been felt, although a decade of repeated and consistent measurement make this unlikely. The Wild Coast region still falls well below provincial and national standards in key areas such as access to clean water and employment. Inequities in access to health services leave the most vulnerable in a continued negative cycle, as poor health impacts negatively on income generating opportunities and increase the burden of health costs for households that are already struggling to survive.

## Competing interests

The authors declare that they have no competing interests.

## Authors' contributions

SM contributed to instrument design, conducted data analysis and drafted the manuscript. NA designed the study, developed the methodology, and contributed to the analysis and the drafting of the manuscript. Both authors have approved the final manuscript.
